# The Arabic version of the sleep hygiene index: Linguistic validation and cultural adaptation among University students in Qatar

**DOI:** 10.5339/qmj.2021.26

**Published:** 2021-09-17

**Authors:** Raja Ali, Monica Zolezzi, Ahmed Awaisu

**Affiliations:** College of Pharmacy, QU Health, Qatar University, Doha, Qatar E-mail: mzolezzi@qu.edu.qa

**Keywords:** sleep hygiene, Arabic, linguistic validation, cultural adaptation, insomnia

## Abstract

Introduction: Sleep is necessary for maintaining adequate health and well-being. However, several behavioral and environmental factors, known as sleep hygiene, could affect sleep quality, contributing to the development of insomnia. Instruments to measure sleep hygiene such as the sleep hygiene index (SHI) have been developed, but only a few are available in the Arabic language. Therefore, this study was designed to translate and culturally adapt the SHI from English to Arabic. Methods: The SHI was translated from English to Arabic using the forward–backward translation method recommended by the International Society for Pharmacoeconomics and Outcomes Research guidelines. Nine Arabic-speaking individuals of diverse educational and cultural backgrounds reviewed the Arabic SHI. The internal consistency reliability of the items contained in the Arabic SHI was determined. Results: The SHI was successfully translated and culturally adapted into the Arabic language. Minimal changes in the wording of some questions were required to ensure the cultural adaptability of the instrument. The Arabic version of the SHI was found to be simple, clear, and brief as reported by the participants during cognitive debriefing. The Arabic SHI has moderate internal consistency reliability with a Cronbach's alpha of 0.589. Conclusion: The Arabic SHI is a brief and easy-to-understand instrument for assessing sleep hygiene practices and behavior in Arabic-speaking population. Further assessment of the psychometric properties of the Arabic SHI is necessary to ensure the validity of this instrument in different populations.

## Introduction

Sleep is a fundamental need for all living organisms and is critical for maintaining physical and mental health. It is also necessary for maintaining cognitive functions, including learning, memory, and performance of complex mental tasks. Adults need around seven to nine hours of sleep per night to preserve their health.^1^ It is well established that sleep can be compromised by surrounding environmental conditions and some behavior that can negatively affect a healthy sleep pattern or sleep quality. The set of behavioral and environmental factors that promote healthy sleep is referred to as “sleep hygiene.”^2^ Inadequate sleep hygiene was identified as one of the causes of insomnia in the second edition of the International Classification of Sleep Disorders (ICSD-2).^3^ Negative sleep hygiene practices highlighted in the ICSD-2 include those which act by promoting arousal or disturbing the normal balance of the sleep–wake cycle.^3^ Furthermore, previous studies suggested that sleep hygiene practices have a significant influence on sleep quality and duration, and they found an increased risk of developing insomnia in individuals with poor sleep hygiene practices.^4–7^ Several factors including irregular sleep schedules and frequent stimulant consumption such as caffeine and energy drinks, especially around bedtime and daytime napping, contribute to worsening sleep problems.^8–16^ Activities in bed, such as reading or watching television, have also been associated with subjective measurements of poor sleep.^17^

These observations highlight the importance of assessing sleep hygiene practices in individuals presenting with insomnia. Therefore, several instruments have been developed to identify individuals with poor sleep hygiene practices. In a recently published systematic review of the literature, four instruments that evaluate sleep hygiene behavior and practices in adults were identified, namely, the Sleep Hygiene Awareness and Practice Scale (SHAPS), the Sleep Hygiene Self-Test (SHS), the sleep hygiene index (SHI), and the Sleep Practices and Attitudes Questionnaire (SPAQ). The major identified drawback of some of these instruments was their length (e.g., the SPAQ consists of 150 questions), and some appear to have been developed without a clear rationale for the item selection (e.g., SHAPS and SHS).^18^ However, the SHI was generated from the diagnostic criteria for inadequate sleep hygiene as defined in the ICSD-2.^3^ The SHI consists of 13 items that evaluate the frequency of engagement in practices and behavior that compromise sleep quality or duration, and it utilizes a simple and practical score calculation strategy.^17^ When its developers first validated the instrument, it showed moderate internal consistency reliability (α = 0.66) and good test–retest stability (*r* = 0.71; *p* < 0.001), and the total score was positively associated with sleep quality and daytime sleepiness.^17^ The SHI has also been translated into at least five languages (Persian, Turkish, Korean, Brazilian-Portuguese, and Indonesian^19–23^) and validated in different populations including university students, the elderly, and individuals with chronic pain.^6,21,24^

Several studies have assessed the prevalence of insomnia among university students in Arab countries, with a reported prevalence ranging 8–85%.^25–31^ Most sleep complaints in this population are related to difficulties in initiating or maintaining sleep and having an interrupted sleep pattern.^32–34^ Sleep insufficiency is also common in this population, with sleep duration not exceeding 6.5 hours.^25,31^ Studies have revealed that irregular sleeping patterns between weekdays and weekends were common among Arab university students, with some students reporting attending university on some weekdays without sleeping.^15,33^ However, these studies did not evaluate sleep hygiene, possibly due to the lack of validated and culturally adapted sleep hygiene instruments for use among Arabic-speaking populations. The cultural adaptability of an instrument is important to ensure that its translated version is linguistically and conceptually equivalent to the original instrument.^35^ Additionally, at the time of conducting this study, no Arabic version of the SHI was available; therefore, this study aimed to translate the SHI from English to Arabic and culturally adapt the instrument for use among Arabic-speaking populations.

## Methods

### Description of the SHI

The English SHI version is provided in Table 1. The SHI consists of 13 self-rated questions with a response option in a 5-point Likert-type scale, ranging between 0 (Never) to 4 (Always), with the total score generated through summation of each item score. The maximum obtainable score of the SHI is 52, such that higher scores indicate worse sleep hygiene.^17^ Although the instrument itself does not provide cut off scores, a study found that a total cutoff score of 16 on the SHI had the best sensitivity (77.0%) and specificity (47.5%) to identify students who were categorized as experiencing poor sleep quality.^6^

The SHI was translated to Arabic following the forward–backward translation method recommended by the International Society for Pharmacoeconomics and Outcomes Research (ISPOR) guidelines.^36^ Before starting the instrument translation process, permission was obtained from the tool developer (Dr. David Mastin^17^). Three independent translators who were native Arabic language speakers translated the SHI from English to Arabic. One of whom was a professional translator with experience in translating documents from English to Arabic. The second was a clinical pharmacist with experience in mental health, and the third was an individual experienced in translation at the College of Pharmacy, Qatar University (QU). A fourth individual who is a healthcare professional (pharmacist) conducted the reconciliation of the resultant three Arabic translations of the SHI, producing the first reconciled Arabic translation of the SHI.

To ensure the accuracy of the Arabic translation, two individuals with background in healthcare translated independently the first reconciled SHI from Arabic back to English. Following that, the backward translation was reviewed in a meeting, which was attended by the backward translators, research team, and the translator who developed the first reconciled Arabic version of the instrument. In this meeting, the two backward translations were compared with the original English version of the SHI, and when any discrepancy was identified, the Arabic version of the instrument was reviewed and edited. At the end of the meeting, the second reconciled Arabic version of the instrument was developed.

A cognitive debriefing process was undertaken to assess the clarity and appropriateness of the content, time burden, and acceptability of the translated SHI among a sample of lay people who could read and write in Arabic, consistent with the variability of the Arabic language speakers living in Qatar. This step was important to identify misunderstandings, inconsistent interpretations, or incomplete concept coverage.^37^ A convenient sample of 10 individuals were interviewed for the cognitive debriefing step; nine of whom were native Arabic language speakers, and one spoke Arabic fluently as a second language. The selected participants reflected different age groups, occupations, nationalities, and cultural backgrounds (Table 2). The following questions were asked during the cognitive debriefing interviews:

a. Can you comment on the Arabic title of the instrument? Is it understandable? Do you have any recommendations?
b. Can you comment on each of the 13 questions on the instrument? Are they clear? Do you have any recommendations to improve their clarity (e.g., changing any vague wording or simplifying a question that was found difficult to understand)?
c. Can you comment on the response options? Are they clear? Do you have any recommendations to improve their clarity (e.g., changing any vague wording or simplifying a response option that was found difficult to understand)?

After the cognitive debriefing was completed, the final version of the Arabic SHI was developed with response options ranging from 0 (Never = أبدا) to 4 (Always = دائما). The translated instrument is presented in Table 3.

### Participants

Students from all colleges enrolled at QU at the time of conducting the study (January 7–March 7, 2019), who could read and write English and/or Arabic, were eligible for participation. After accounting for a response rate of 15%, it was determined that the survey had to be sent to 2200 students to obtain the required sample size of 325. The original plan was to select the 2200 students using a proportionate, stratified simple random sampling technique. However, the minimum sample size required was not achieved when this approach was followed; therefore, the survey was sent to all QU students. Students were recruited for participation in the study through invitation emails. The survey containing the SHI was built in an online platform using SurveyMonkey®. The survey was sent to the participants in English and Arabic; however, to assess the reliability of the Arabic translation of SHI, only the responses on the Arabic SHI were included. For the purposes of this study, participants were required to provide responses in relation to their sleep hygiene practices in the previous month.

The participants completed consent forms before participating in the study. All responses were anonymous. Qatar University Institutional Review Board approved the study (reference number of: QUIRB 977-EA/18).

### Reliability testing

Data analysis was conducted using the Statistical Package for Social Sciences (SPSS) software version 26 (IBM SPSS^®^ Statistics for Windows, IBM Corp, Armonk, New York, USA). The reliability of the Arabic version of the SHI was assessed using Cronbach's alpha, a measure of reliability that assesses the internal consistency of scale items.

## Results

The Arabic version of the SHI was found to be simple, clear, and brief (Table 3). The title, instructions, and response options of the Arabic SHI were found to be understandable to a lay person (i.e., with enough education to read and understand Arabic) as reported by the participants. However, several changes were deemed necessary during the translation and cognitive debriefing processes to ensure cultural adaptability and acceptability of the instrument. A summary of the modifications implemented is presented as follows:

• Response option 3: “in a frequent way”: Some participants recommended rewording response option number 3 from “in a frequent way = بشكل متكرر” to a synonym that translates to “frequently = غالبا.”
• Item 5: “I stay in bed longer than I should two or three times a week”: Some respondents found this question to be confusing as it was unclear if the question was inquiring about the duration they spend in bed asleep or after waking up in the morning. Therefore, the developer of the original instrument was contacted to obtain further clarification about the meaning of this question. Based on the response, the question was modified by adding in brackets “after waking up” to eliminate confusion and clarify the question.
• Item 6: “I use alcohol, tobacco, or caffeine within 4 hr of going to bed or after going to bed”: During the translation process, it was recommended to eliminate the word “alcohol” to ensure the cultural acceptability of the question and minimize the nonresponse rate to this question. It was also recommended to add examples of caffeine-containing beverages to ensure the clarity of this question for people with varying education levels.
• Item 9: “I use my bed for things other than sleeping or sex”: The translators found the word “sex” to be culturally inappropriate and was, therefore, replaced with “marital intimacy.”

The Arabic SHI was used in the study population after the above changes were implemented. The SHI score was calculated for the 1588 students who responded to the Arabic version of the SHI. The demographics of the respondents are presented in Table 4. The internal consistency reliability of the SHI items using Cronbach's alpha coefficient was 0.589. Deleting items from the scale was associated with minimal changes in Cronbach's alpha values. However, deletion of either item 1 or 5 was associated with an increase in Cronbach's alpha values to 0.603 and 0.604, respectively (Table 5), indicating that removing these items improves the internal consistency of the Arabic SHI. Interestingly, deleting item 2 “I go to bed at different times from day to day” did not change the Cronbach's alpha value. Meanwhile, deleting any other remaining item was associated with lower Cronbach's alpha values, indicating that removing these items decreases the internal consistency of the Arabic SHI.

## Discussion

The objective of the study was achieved as the Arabic version of the SHI was developed and culturally adapted for use among the Arab-speaking population. From the four sleep hygiene instruments available, the SHI was selected because of its advantages over other tools.^18^ Compared with other instruments, SHI was found to be the simplest in terms of the language, item numbers, and response options. Additionally, the SHI was developed in accordance with the ICSD-2 criteria of inadequate sleep hygiene and is the only sleep hygiene instrument for adults to be translated into various languages and validated among diverse populations.^3^

To ensure achieving a rigorous and accurate translation of the instrument, the ISPOR criteria were followed in the translation process. Three Arabic-native translators were involved in the forward translation step; they came from different backgrounds, which helped provide more insight into the translation. The first translator was a professional who was well accustomed to the commonly used language, whereas the second was a clinical pharmacist who is knowledgeable in terminologies used in practice. The third translator was experienced in conducting translations within the academic field. Each translator independently came with the first drafts of Arabic SHI, and these three versions were then reconciled by a fourth translator who was a clinical practitioner native in the Arabic language. The involvement of four individuals coming from diverse fields in the translation has helped in ensuring that the developed translation of the instrument is understandable from the perspectives of lay individuals and people in the healthcare field. The inclusion of more than one translator in each stage provided a mix of perspectives and, thereby, minimized individual translator's bias, which could affect the equivalence with the original instrument.^37^

Establishing the cultural adaptability of the SHI was necessary to ensure the clarity of the questions to the target audience. Additionally, the adaptation facilitates the investigation of cross-cultural differences using the instrument's results. Cross-cultural adaptation is important to ensure that the translated instrument measures the same concepts as in the original instrument. A culturally adapted instrument is a tool that is conceptually and linguistically equivalent to the source language as well as being culturally competent and linguistically appropriate for the target population.^35^ The participants involved in the pilot testing (cognitive debriefing process) were generally satisfied with the title of the instrument and found the questions to be clear and understandable. Recently, Costa et al.,^38^ reported another Arabic version of the SHI. However, the researchers used professional translators and project managers to produce the Arabic version of the SHI and did not report pilot testing or cultural adaptation processes. Considering that this study's Arabic version of the SHI was pilot tested among 10 Arabic speakers of different nationalities and cultural backgrounds supports the cultural adaptability of the translated SHI.

A few modifications were required in the Arabic version of the SHI, and most of which were minor changes. The translators raised the issue of cultural appropriateness during the forward translation of the SHI and involved removing the word “alcohol” from question 6 and replacing the word “sex” in question 9 with “marital intimacy.” It was suggested that question 6 in its original form could be problematic as it lists alcohol with tobacco and caffeine while asking about the frequency of use; alcohol consumption is prohibited in Islam and is not common among the Arabic-speaking populations. So, there were fears that the participants might decline to answer this question or could provide an answer not reflecting their actual behavior (i.e., consumption of caffeine or tobacco). It was also recommended to include examples of caffeinated beverages in this question to clarify it further for all respondents. For question 9, “marital intimacy” was found to be a more appropriate expression in the Arabic culture. During the cognitive debriefing, the participants found question 5 “I stay in bed longer than I should two or three times a week” to be confusing, as it was unclear if the question was referring to the time spent in bed asleep or awake in the morning. After clarification, it was found that the question referred to the time spent in bed after waking up in the morning and was modified thereafter.

The Arabic SHI was shown to have a moderate internal consistency with Cronbach's alpha of 0.59, which is lower than the reported Cronbach's alpha for the Turkish (0.70 and 0.71), Korean, and Brazilian-Portuguese (0.75 for each) versions of the instruments; however, it was close to the internal consistency of the Indonesian (0.618) and English (0.66 and 0.64) versions of the SHI.^17^,^19^,^36^–^38^ Recently, Costa et al.,^38^ reported a Cronbach's alpha value of 0.749 for their Arabic translation of the SHI. The differences may be partially due to the differing populations in which the SHI was administered in the two studies (general population vs. university students), Costa et al., version's lack of cultural adaptation of the translation, or the time when the instrument was validated. Costa et al., reported that their instrument was administered during the coronavirus 2019 quarantine period, when the ritual sleep hygiene behavior could have been altered.

The similarity between the Cronbach's alpha values for this study's sample to those reported for the English and Indonesian versions of the instrument could be related to the fact that these instruments were validated among university students and adolescents, respectively. On the other hand, studies that included clinical samples such as individuals with chronic pain, depression, and adult hospital workers (in the Korean, Turkish, and Brazilian-Portuguese versions of SHI, respectively) reported higher Cronbach's alpha values. Similarly, the individuals included in the study of Costa et al., were older than those in this study, as it was administered in the general population in Lebanon.^38^ Therefore, the difference in the observed internal consistency might be partly derived from the differences in the validated sample characteristics.

Since the SHI questions were developed from the ICSD-2 definition of maladaptive sleep hygiene, which consists of several components not directly related but that affect sleep sleep negatively, this might explain the lower internal consistency observed. However, the lower Cronbach's alpha value observed for this study's instrument does not affect the utility of the tool, as the main objective of an instrument is to produce an interpretable score, and this is possible even when the alpha value is not high.^39^

Assessing other psychometric properties of this instrument, including reproducibility and validity measures, was not possible in this study because of time restrictions. Therefore, it is recommended for future studies to assess the test–retest reliability and validity measures of the Arabic SHI. Investigating the interpretability of the instrument would also be useful to identify cutoff scores to distinguish between good and poor sleep hygiene practices. Additionally, there is a need to validate the Arabic SHI among different populations and subgroups, including both healthy individuals and those experiencing clinical conditions, which can be affected by varying patterns of sleep hygiene or which could be the cause of these irregular sleep hygiene practices. It is also recommended to compare this study's results with those obtained for the same individuals using a different sleep hygiene instrument available in Arabic.

Another limitation that may need to be explored in future research is the changes made to the SHI during the cultural adaptation process (e.g., the words “marital intimacy” were used instead of “sex,” and the word “alcohol” was removed). Although these changes were consulted and accepted by the developer of the SHI, they need to be further explored and compared with other translations of the instrument that were not culturally adapted. Considering the recently published Arabic version reported by Costa et al., ^38^ provides an opportunity to assess their comparative properties so that a consolidated Arabic version is considered for use in the future.

## Conclusion

This Arabic and culturally adapted version of the SHI is a short, simple, and easy to comprehend instrument, which is suitable for administration to the average literate individuals in the Arabic language. The SHI items have an acceptable internal consistency; however, further exploration of the psychometric properties of the Arabic SHI is necessary.

## Disclosure

This research was supported by a student grant from Qatar University (QUST-1-CPH-2019-5). The authors declare that they have no conflict of interest to disclose.

## Acknowledgments

We would like to thank Dr. Fatima Mraich, Dr. Yassin El-torki, Ms. Huda Barhoosh, and Pharmacist Mohamed Mostafa Moursi for their involvement in the translation of the sleep hygiene index to the Arabic language. We also thank all individuals who have participated in this study and answered the survey questions.

## Figures and Tables

**Table 1:** Sleep Hygiene Index (edited version following cultural adaptation)

Below you will find a list of statements. Please rate how true each statement is for you by circling a number next to it				

0	1	2	3	4

Never	Rarely	Sometimes	Frequent	Always

1. I take daytime naps lasting two or more hours.				

2. I go to bed at different times from day to day.				

3. I get out of bed at different times from day to day.				

4. I exercise to the point of sweating within 1 hour of going to bed.				

5. I stay in bed longer than I should (after waking up)^a^ two or three times a week.				

6. I use tobacco or caffeine (for example: coffee or tea)^a^ within 4 hours of going to bed or after going to bed.				

7. I do something that may wake me up before bedtime (for example: play video games, use the internet, or clean).				

8. I go to bed feeling stressed, angry, upset, or nervous				

9. I use my bed for things other than sleeping or marital intimacya (for example: watch television, read, eat, or study).				

10. I sleep on an uncomfortable bed (for example: poor mattress or pillow, too much or not enough blankets).				

11. I sleep in an uncomfortable bedroom (for example: too bright, too stuffy, too hot, too cold, or too noisy).				

12. I do important work before bedtime (for example: pay bills, schedule, or study).				

13. I think, plan, or worry when I am in bed.				


^a^ Edited during the cultural adaptation process

**Table 2:** Cognitive debriefing: Participant demographics

Participant	Gender	Age (years)	Nationality	Occupation

1	Female	28	Pakistani	Unemployed

2	Male	53	Djiboutian	Researcher

3	Female	47	Yemeni	Teacher

4	Female	24	Egyptian	Student

5	Female	27	Sudanese	Pharmacist

6	Female	24	Iraqi	Pharmacist

7	Female	19	Syrian	Student

8	Female	16	Qatari	Student

9	Female	17	Djiboutian	Student

10	Female	24	Egyptian	Student


**Table 3:**
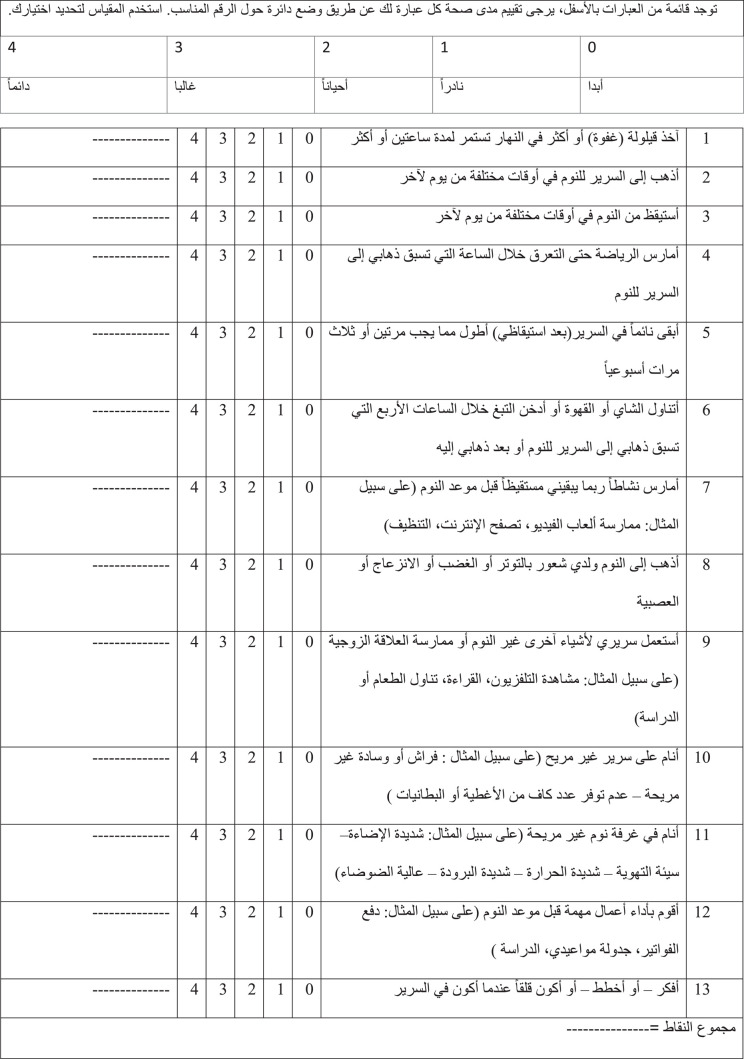
Arabic version of the sleep hygiene index 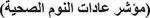

**Table 4:** Demographic characteristics of survey respondents (n = 1574*)

Variable	n (%)

Gender	

Male	228 (14.5)

Female	1346 (85.5)

Age category (years)	

≤23	1084 (68.9)

>23	490 (31.1)

Marital status	

Single	1233 (78.34)

Married	341 (21.66)

College	

Arts and Sciences	519 (32.9)

Business and economics	303 (19.3)

Education	179 (11.4)

Engineering	242 (15.4)

Health sciences	58 (3.7)

Law	122 (7.8)

Medicine	27 (1.7)

Pharmacy	26 (1.6)

Sharia and Islamic studies	98 (6.2)

*Missing data = 14 did not complete the demographics section but completed the section related to the sleep hygiene index (Arabic version).	


*Missing data = 14 did not complete the demographics section but completed the section related to the sleep hygiene index (Arabic version).

**Table 5:** Cronbach’s alpha for items of the Arabic SHI (N = 1588)

Item	Score mean if item is deleted^*^	Score variance if item is deleted	Cronbach’s alpha if item is deleted

SHI 1	19.4	36.6	0.603

SHI 2	19.2	32.7	0.589

SHI 3	18.6	30.4	0.54

SHI 4	18.8	31	0.55

SHI 5	20	35.8	0.604

SHI 6	18.8	30.2	0.545

SHI 7	19.5	31.3	0.571

SHI 8	17.9	30.7	0.551

SHI 9	19.1	31.1	0.552

SHI 10	18.5	30.3	0.563

SHI 11	20.2	33.6	0.579

SHI 12	20.2	32.7	0.568

SHI 13	18.8	31.8	0.572


*The score for each item ranges between 0 (never) and 4 (always), whereas the total score ranges between 0 and 52.

SHI, sleep hygiene index.

## References

[bib1] Hirshkowitz M, Whiton K, Albert SM, Alessi C, Bruni O, DonCarlos L (2015;). National Sleep Foundation's sleep time duration recommendations: methodology and results summary. Sleep Health.

[bib2] Irish LA, Kline CE, Gunn HE, Buysse DJ, Hall MH (2015;). The Role of Sleep Hygiene in Promoting Public Health: A Review of Empirical Evidence HHS Public Access. Sleep Med Rev.

[bib3] American Academy of Sleep Medicine The International Classification of Sleep Disorders (ICSD), Revised. Diagnostic and Coding Manual.

[bib4] Gellis LA, Park A, Stotsky MT, Taylor DJ (2014;). Associations between sleep hygiene and insomnia severity in college students: cross-sectional and prospective analyses. Behav Ther.

[bib5] Brick CA, Seely DL, Palermo TM (2010;). Association between sleep hygiene and sleep quality in medical students. Behav Sleep Med.

[bib6] Seun-Fadipe CT, Aloba OO, Oginni OA, Mosaku KS (2018;). Sleep hygiene index: Psychometric characteristics and usefulness as a screening tool in a sample of Nigerian undergraduate students. J Clin Sleep Med.

[bib7] Suen LK, Tam WW, Hon KL (2010;). Association of sleep hygiene-related factors and sleep quality among university students in Hong Kong. Hong Kong Med J.

[bib8] Gaultney JF (2010;). The prevalence of sleep disorders in college students: impact on academic performance. J Am Coll Heal.

[bib9] Buboltz WC, Brown F, Soper B (2001;). Sleep habits and patterns of college students: a preliminary study. J Am Coll Health Assoc.

[bib10] Li L, Wang YY, Wang SB, Li L, Lu L, Ng CH (2017;). Sleep Duration and Sleep Patterns in Chinese University Students: A Comprehensive Meta-Analysis. J Clin Sleep Med.

[bib11] Haile YG, Alemu SM, Habtewold TD (2017;). Insomnia and its temporal association with academic performance among university students: a cross-sectional study. Biomed Res Int.

[bib12] Elwasify M, Barakat DH, Fawzy M, Elwasify M, Rashed I, Radwan DN (2016;). Quality of sleep in a sample of Egyptian medical students. Middle East Curr Psychiatry.

[bib13] Alsaggaf MA, Wali SO, Merdad RA, Merdad LA (2016;). Sleep quantity, quality, and insomnia symptoms of medical students during clinical years: relationship with stress and academic performance. Saudi Med J.

[bib14] Tannous M, Al Kalash Y (2014;). Prevalence of caffeinated-beverage consumption by university students in North Lebanon. Public Heal Res.

[bib15] Satti GMH, Alsaaid HF, Nabil NM, Saeed AA, Alhamdan N, El-bakri NK (2015;). The prevalence of sleep problems and its impact on sleep quality and academic performance. Merit Res J Educ Rev.

[bib16] Ibrahim NK, Iftikhar R, Murad M, Fida H, Abalkhaeil B, Al Ahmadi J (2014;). Energy drinks consumption amongst medical students and interns from three colleges in Jeddah, Saudi Arabia. J Food Nutr Res.

[bib17] Mastin DF, Bryson J, Corwyn R (2006;). Assessment of sleep hygiene using the sleep hygiene index. J Behav Med.

[bib18] Ali RM, Awaisu A, Zolezzi M (2020). A systematic review of instruments assessing insomnia and sleep hygiene practices among adults. Nat Sci Sleep.

[bib19] Chehri A, Kiamanesh A, Ahadi H, Khazaie H (2016;). Psychometric properties of the persian version of sleep hygiene index in the general population. Iran J Psychiatry Behav Sci.

[bib20] Ozdemir PG, Boysan M, Selvi Y, Yildirim A, Yilmaz E (2015;). Psychometric properties of the Turkish version of the sleep hygiene index in clinical and non-clinical samples. Compr Psychiatry.

[bib21] Cho S, Kim GS, Lee JH (2013;). Psychometric evaluation of the sleep hygiene index: a sample of patients with chronic pain. Health Qual Life Outcomes.

[bib22] Tonon AC, Amando GR, Carissimi A, Freitas JJ, Xavier NB, Caumo GH (2020;). The Brazilian-Portuguese version of the Sleep Hygiene Index (SHI): validity, reliability and association with depressive symptoms and sleep-related outcomes. Sleep Sci.

[bib23] Setyowati A, Chung MH, Yusuf A, Haksama S (2020;). Psychometric properties of sleep hygiene index in indonesian adolescents. J Public health Res.

[bib24] Chehri A, Parsa L, Khazaie S, Khazaie H, Jalali A (2020;). Validation of the sleep hygiene index for the elderly. J Public Heal.

[bib25] Sweileh WM, Ali IA, Sawalha AF, Abu-Taha AS,  Zyoud SH, Al-Jabi SW (2011;). Sleep habits and sleep problems among Palestinian students. Child Adolesc Psychiatry Ment Heal.

[bib26] Al-Eisa E, Buragadda S, Melam GR, Al-Osaimi AO, Al-Mubarak HA, Al-Huwaimel NA (2013;). Association between physical activity and insomnia among Saudi female college students. J Phys Ther Sci.

[bib27] Afandi O, Hawi H, Mohammed L, Salim F, Hameed AK, Shaikh RB (2013;). Sleep Quality Among University Students : Evaluating the Impact of Smoking, Social Media Use, and Energy Drink Consumption on Sleep Quality And Anxiety. Inq Journal/Student Pulse.

[bib28] Alqudah M, Balousha SAM, Al-Shboul O,  Al-Dwairi A, Alfaqih MA, Alzoubi KH (2019;). Insomnia among medical and paramedical students in Jordan: impact on academic performance. Biomed Res Int.

[bib29] Kabrita CS, Hajjar-Muça TA, Duffy JF (2014;). Predictors of poor sleep quality among Lebanese university students: association between evening typology, lifestyle behaviors, and sleep habits. Nat Sci Sleep.

[bib30] Assaad S, Costanian C, Haddad G, Tannous F (2014;). Sleep patterns and disorders among university students in Lebanon. J Res Health Sci.

[bib31] Suleiman K, Yates B, Jassem H, Alghabeesh S, Luai Abu Shahroor, Ali R (2013;). Sleep disturbances among Alzaytoonah university students in Jordan. J Nat Sci Res.

[bib32] Abdel–Khalek AM (2006;). Prevalence of insomnia complaints and its conseuqneces in Kuwaiti college students. Sleep Hypn.

[bib33] Abdeen ZA, Jadallah AS, Al-qahtani MH,  Albashbishi HA, Hamadeh RR (2013;). Sleeping patterns among Arabian Gulf university medical students. Arab Gluf J Sci Res.

[bib34] Abdel–Khalek A, Al-Nyal M, Saeed H (2014;). Insomnia among Egyptian samples of university students and employees. J Educ Psychol Stud Sultan Qaboos Univ.

[bib35] Weech-Maldonado R, Weidmer BO, Morales LS, Hays RD (2001:). Cross-cultural adaptation of survey instruments: The CAHPS experience. In: *Seventh Conference on Health Survey Research Methods*.

[bib36] Wild D, Grove A, Martin M, Eremenco S, McElroy S, Verjee-Lorenz A (2005;). Principles of Good Practice for the Translation and Cultural Adaptation Process for Patient-Reported Outcomes (PRO) Measures: report of the ISPOR Task Force for Translation and Cultural Adaptation. Value Heal.

[bib37] Epstein J, Santo RM, Guillemin F (2015;). A review of guidelines for cross-cultural adaptation of questionnaires could not bring out a consensus. J Clin Epidemiol.

[bib38] Costa J, Helou S, Sleilaty G, Costa T, El Helou J (2021;). Validity and reliability of an Arabic version of the Sleep Hygiene Index. Sleep Med.

[bib39] Taber KS (2018;). The use of Cronbach's alpha when developing and reporting research instruments in science education. Res Sci Educ.

